# Improvement of Schottky Contacts of Gallium Oxide (Ga_2_O_3_) Nanowires for UV Applications

**DOI:** 10.3390/s22052048

**Published:** 2022-03-06

**Authors:** Badriyah Alhalaili, Ahmad Al-Duweesh, Ileana Nicoleta Popescu, Ruxandra Vidu, Luige Vladareanu, M. Saif Islam

**Affiliations:** 1 Nanotechnology and Advanced Materials Program, Kuwait Institute for Scientific Research, P.O. Box 24885, Kuwait City 13109, Kuwait; bhalaili@kisr.edu.kw (B.A.); ahmed.alduweesh@gmail.com (A.A.-D.); 2 Faculty of Materials Engineering and Mechanics, Valahia University of Targoviste, 130004 Targoviste, Romania; pinicoleta24@yahoo.com; 3 Faculty of Materials Science and Engineering, University Politehnica of Bucharest, 060042 Bucharest, Romania; 4 Department of Electrical and Computer Engineering, University of California, Davis, Davis, CA 95616, USA; sislam@ucdavis.edu; 5 Robotics and Mechatronics Department, Institute of Solid Mechanics, Romanian Academy, 030167 Bucharest, Romania

**Keywords:** Ga_2_O_3_, quartz, nanowires, metal contacts

## Abstract

Interest in the synthesis and fabrication of gallium oxide (Ga_2_O_3_) nanostructures as wide bandgap semiconductor-based ultraviolet (UV) photodetectors has recently increased due to their importance in cases of deep-UV photodetectors operating in high power/temperature conditions. Due to their unique properties, i.e., higher surface-to-volume ratio and quantum effects, these nanostructures can significantly enhance the sensitivity of detection. In this work, two Ga_2_O_3_ nanostructured films with different nanowire densities and sizes obtained by thermal oxidation of Ga on quartz, in the presence and absence of Ag catalyst, were investigated. The electrical properties influenced by the density of Ga_2_O_3_ nanowires (NWs) were analyzed to define the configuration of UV detection. The electrical measurements were performed on two different electric contacts and were located at distances of 1 and 3 mm. Factors affecting the detection performance of Ga_2_O_3_ NWs film, such as the distance between metal contacts (1 and 3 mm apart), voltages (5–20 V) and transient photocurrents were discussed in relation to the composition and nanostructure of the Ga_2_O_3_ NWs film.

## 1. Introduction

Wide bandgap-based semiconductors are suitable for harsh environmental applications, such as in UV photodetectors, especially high-powered electronics and optoelectronics that operate in particularly harsh environmental applications, such as solar UV monitoring, communications, and the detection of missiles [1,2,3,4]. Accordingly, different studies have focused on the fabrication of UV sensors that can respond to and withstand harsh environments while remaining blind to visible wavelengths. The scientific research community has been working to explore wide bandgap materials that could be effective for use in UV photodetectors, such as ZnO [5,6,7] and Ga_2_O_3_ [2,3]. 

The Ga_2_O_3_-based semiconductor is one of the most promising materials for UV detection, power electronics, and solar-blind UV photodetectors [2,3]. Β-Ga_2_O_3_ is the most chemically and thermally stable monoclinic structure [8]. In addition, β-Ga_2_O_3_ has a wide bandgap of 4.9 eV, a high melting point of 1900 °C, excellent electrical conductivity, and photoluminescence [8]. Despite these interesting properties, it has only been in recent years that interest in this material has grown.

Recently, investigators have shown interest in the fabrication of low-dimensional β-Ga_2_O_3_ nanowires (NWs) because of their exceptional properties. Ga_2_O_3_ nanowires have been proposed for many applications, such as optical and sensing studies [9,10]. The use of Ga_2_O_3_ nanowires has shown significant improvements in responsivity due to their large surface-to-volume ratio, small dimension, light confinement [11], and high gain in photoconductivity [12]. In this work, an inexpensive thermal oxidation process at 1000 °C was performed to grow β-Ga_2_O_3_ nanowires for UV photodetection by using an Ag catalysts in the presence of a few oxygen molecules. 

The results of morphological, structural, electrical, and optical characterizations of the β-Ga_2_O_3_ nanowires have resulted in the development of optical bandgap and photoconductance sensors, with multiple applications in the development of intelligent real-time control interfaces of robots. The significant interest in UV photodetectors (PDs) lately is highlighted by the need for improved UV instruments that operate in extremely harsh environments with a high impact on civilian, military, and robotics technologies, in addition to soft robotics applications [12,13]. Recent advances in the field of soft robotics and wearables have increased the demand for printed soft and flexible electronics, additive manufacturing techniques, etc. [14,15].

Alhalaili et al. [16] have shown that both the nucleation and growth morphology of Ga_2_O_3_ nanostructure film obtained by thermal oxidation depend on several factors, such as the substrate on which Ga_2_O_3_ is grown, the use of Ag catalyst, and the oxidation temperature. The morphological and elemental analyses of Ga_2_O_3_ nanostructures indicated an increase in the density that the Ga_2_O_3_ nanowires obtained in the presence of the Ag catalyst. Moreover, when Ag was used, the coverage of Ga_2_O_3_ NWs obtained at 1000 °C was very broad, with morphological features including long and thin nanowires with sharp tips for various electronic and optoelectronic applications.

Various challenges were identified at the substrate/Ga_2_O_3_ interface [16], which require further investigation to better control the growth mechanism and morphology needed to produce uniform films of Ga_2_O_3_ nanostructures. When the morphologies of the nanowires grown on bare quartz and Ag-coated quartz were compared, it was found that the Ag catalyst enhanced nanowire growth rates and increased the density of nucleation sites [12]. The Ag catalyst appears to play an important role in the development of nanowires, reducing the diameters of the nanowires, increasing the height, and sharpening the tips. The bandgaps of β-Ga_2_O_3_ nanowires with and without silver were found to be 4.6 eV and 4.4 eV, respectively. The initial I–V measurements of the photocurrent were performed at different selected wavelengths with a 1mW LED lamp [12]. The photodetection response of Ga_2_O_3_ nanostructures grown on Ag-coated quartz increased by almost two orders of magnitude compared to the Ga_2_O_3_ nanostructures grown on bare quartz, while the ratio of photocurrent to dark current was almost 9-times higher, leading to a more sensitive detection of UV light.

In this manuscript, we communicate the first results of the influence of different metal contacts to improve the electrical properties of Ga_2_O_3_ NWs and to study their photoconductance. In addition, we discuss the factors that affect the detection process of Ga_2_O_3_ NWs material, such as the distance between metal contacts, voltages, and the transient photocurrent. 

## 2. Materials and Methods

**Figure 1:**
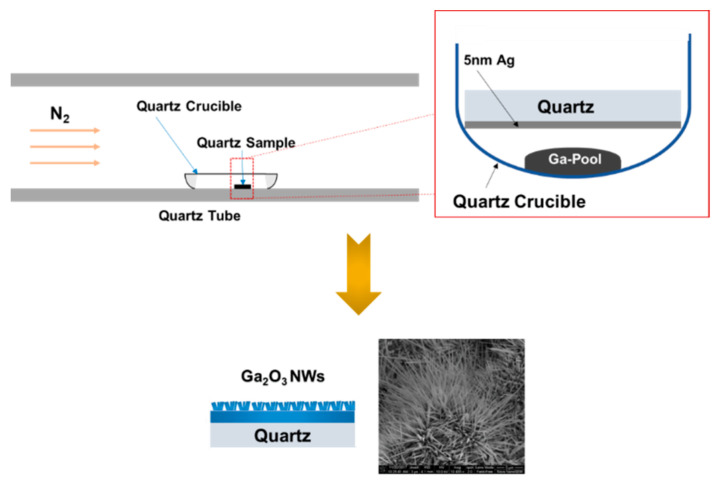
Schematic illustration of the fabrication process of Ga_2_O_3_ nanowires by thermal growth process at 1000 °C in the presence of silver catalyst and liquid Ga, which was placed at the bottom of the quartz crucible.

First, quartz substrates were cleaned with acetone and ethyl alcohol, rinsed with deionized water, and dried with a N_2_ gun. Quartz substrates were 500 μm thick and 15 mm in diameter. Then, a thin film of 5 nm silver was deposited on quartz substrates using a Lesker sputtering system (Jefferson Hills, PA, USA). To obtain Ga_2_O_3_, 0.2 g of gallium (Ga) (purity 99.999%, Sigma Aldrich, Mountain View, CA, USA) was placed in a crucible. The samples were placed above liquid gallium (Figure 1), with the Ag layer directed toward the Ga pool. To avoid contamination, the experiments were performed in separate treatment cycles and dedicated crucibles, regardless of whether or not Ag-coated samples were used. Then, the quartz crucible containing gallium was inserted into a furnace for the oxidation process. 

**Figure 2:**
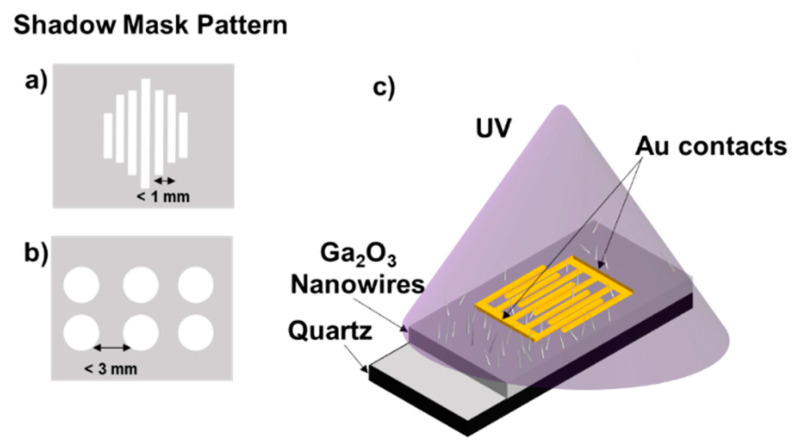
Schematic illustration of different shadow masks (**a**,**b**) used for sputtering 5 nm Cr and 50 nm gold (Au) contacts on Au/β-Ga_2_O_3_/Au metal–semiconductor–metal (MSM) photoconductor on quartz. (**a**) The distance between the lines is <1 mm. (**b**) The distance between the circle probes is <3 mm. (**c**) Schematic illustration of Au/β-Ga_2_O_3_/Au metal–semiconductor–metal (MSM) photoconductor on quartz.

Samples were placed in a quartz crucible and inserted in an OTF-1200X-50-SL horizontal alumina tube furnace made by the MTI Corporation (Richmond, CA, USA). The samples were heated to 1000 °C for 1 h. Heating occurred in 20 sccm nitrogen flow. Background oxygen concertations were determined to range from 100 ppm to 200 ppm [13]. The surface of the Ga_2_O_3_ layer was observed by scanning electron microscopy (SEM) to confirm nanowire formations (FEI Nova NanoSEM430, FEI Company, Hillsboro, OR, USA) and to correlate it with electrical performances. Alhalaili et al. [16] have observed, by SEM, energy dispersive X-ray spectroscopy (EDS), and high resolution transmission electron microscopy (HRTEM) equipped with EDS profile analysis, the formation of high-density, long, thin, and ultra-sharp Ga_2_O_3_ nanowires obtained in the presence of the Ag catalyst at 1000 °C, which increased electron transfers [12,13]. After oxidation, electrical contacts were patterned at the top of the nanowires using a shadow mask, 5 nm Cr and 50 nm Au were sputtered using a Lesker sputtering system [9]. The two different patterns of the shadow mask used in the experiments are presented in Figure 2. For electrical measurements, a custom probe station attached to a Keithley 2400 SMU (Beaverton, OR, USA) was used, and UV illumination was observed by using a Dymax Bluewave 75 UV lamp (280–450 nm) (Dymax Corporation, Torrington, CT, USA) [9].

## 3. Results and Discussion

### 3.1. Morphology

**Figure 3:**
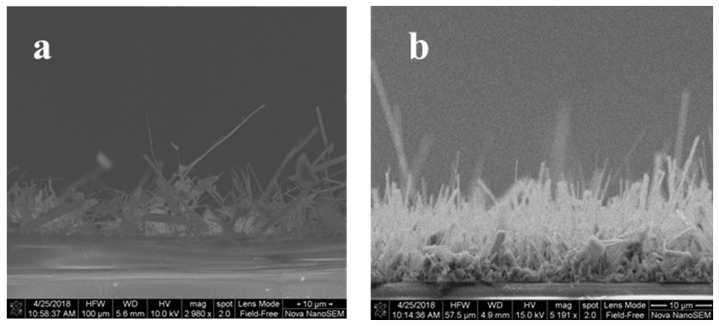
Side views of SEM images of Ga_2_O_3_ nanowire grown at 1000 °C. (**a**) Free-Ag of Ga_2_O_3_ nanowires on a quartz substrate. (**b**) Ga_2_O_3_ nanowires on quartz catalyzed by 5 nm Ag. Longer and denser Ga_2_O_3_ nanowires were attained in the presence of Ag.

The physical, chemical, and structural characterizations of the β-Ga_2_O_3_ nanowire film were presented in detail in our previous work [12]. The morphology of the film presented in Figure 3 shows that the density and length of the nanowires were highly improved when the Ag catalyst was used during the oxidation of Ga. Figure 3a shows the nanowire’s morphology of the layer grown on bare quartz compared to the layer grown in the presence of 5 nm Ag (Figure 3b). The presence of Ag as a catalyst improved the growth of nanowires, resulting in denser, thinner, and longer Ga_2_O_3_ nanowires. The diameter of the nanowires without a silver catalyst is thicker than the one with silver. Table 1 shows a summary of data that compares the growth of Ga_2_O_3_ nanowires with and without the presence of silver catalysts. 

Although the growth of gallium oxide nanowires by thermal oxidation is technically a simple process, the growth mechanism behind spontaneous and rapid formation of nanowires is still not clear. Experimental results have shown that silver nanoparticles play a catalytic role in the growth of Ga_2_O_3_ nanowires during the thermal oxidation of liquid gallium due to the high solubility and diffusivity of O_2_ in Ag [12,16,17,18,19]. The characterization of Ga_2_O_3_ nanowires obtained in the presence of an Ag catalyst as well as their growth mechanism is presented in detail elsewhere [12]. In brief, the growth mechanism is attained by a bottom-up approach, using the dissolved oxygenated gallium species in molten Ga.

At 1000 °C, the diffusion of oxygen molecules increases in the liquid phase, and Ga_2_O_3_ started to nucleate due to the higher affinity liquid Ga has for oxygen than for Ag [12]. Residual oxygen in the chamber reacted with gallium by continuous diffusion to feed the growth of nanowires [20]. During thermal oxidation, a 5 nm Ag ultrathin film catalyzed the growing process of nanowires by providing additional oxygen, which was more than the Ga would be able to absorb on its own [21]. The rate of oxygen adsorption in the presence of the Ag catalyst increased by increasing temperatures [22]. As oxygen was adsorbed on the surface of the Ag catalyst, oxygen may have diffused by volume diffusion or may have desorbed as O_2_ [12]. When silver reached its melting point (961.8 °C) and formed droplets by dewetting from the surface, the adsorption of oxygen increased in the liquid (Ag, Ga) phase. As a result, when the temperature increased, the diffusion length of Ga increased, which caused Ga atoms to spread to Ag [23]. Concomitantly, more oxygen molecules diffused into Ag atoms. Due to the mass transport mechanism, nanowire oxide growth formed a sharp tip. The Ga_2_O_3_ nanowires’ growth proceeded by surface diffusion along the sidewalls of the nanowires from the base to the tip. The diffusion flux of Ga atoms was driven by the chemical potential gradient due to the large difference in oxygen partial pressure between Ga_2_O_3_/air and Ga_2_O_3_/Ga interfaces. However, the chemical and physical interactions between Ga and O are not clearly discussed in the literature. Generally, the solubility of oxygen increases in liquid Ga with increasing Ag nanoparticles [24]. In the three-component system of O-Ag-Ga, the incorporation of Ag nanoparticles into liquid Ga results in greater accessibility of oxygen in the system, which interacts with Ga and Ag nanoparticles to develop longer and denser Ga_2_O_3_ nanowires. As a result, gallium oxide nanowires continue to develop because both the diffusivity and solubility of oxygen increase with increasing temperature [12].

### 3.2. Photocurrent and Dark Current Measurements

#### 3.2.1. Voltage 

**Figure 4:**
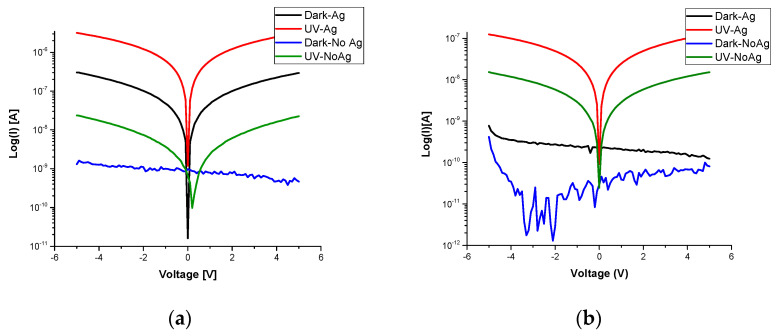

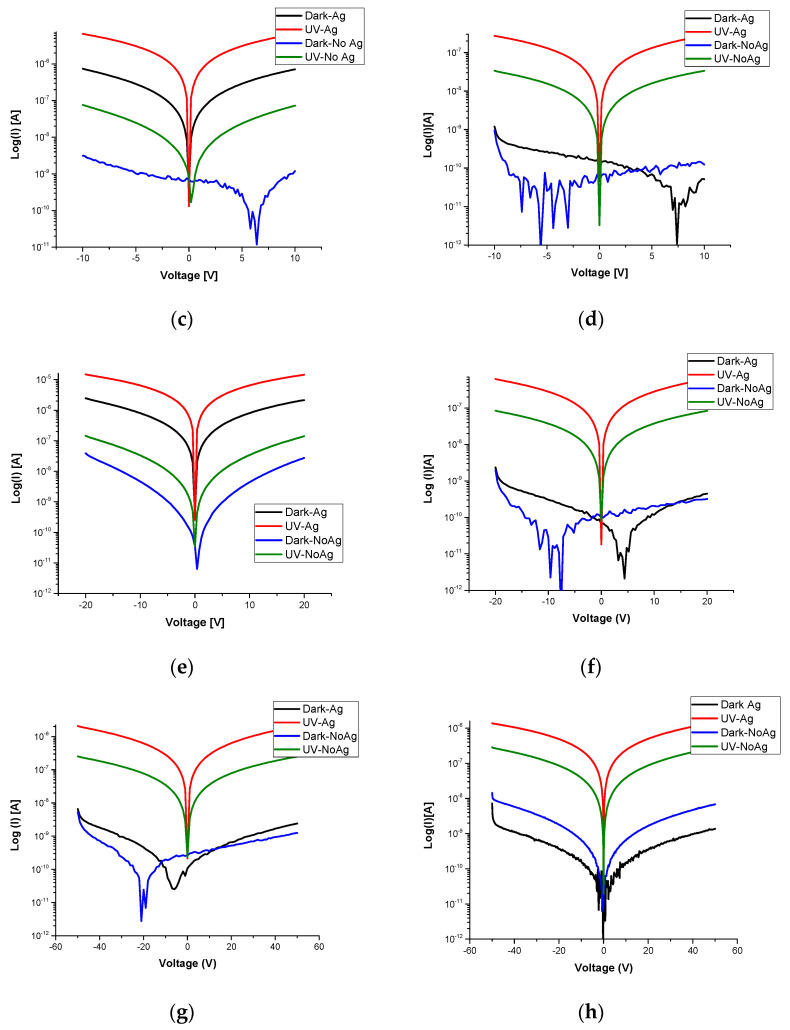
Semi-logarithmic plots of current density for Au/β-Ga_2_O_3_/Au MSM without and with Ag catalyst versus applied voltage characteristics without and with UV illumination. The right column is for the distance between the probe lines <1 mm. Left column is for the distance between the probe lines <3 mm. (**a**,**b**) 5 V. (**c**,**d**) 10 V. (**e**,**f**) 20 V. (**g**,**h**) 50 V.

The fabrication process of the Au/β-Ga_2_O_3_/Au metal–semiconductor–metal (MSM) photoconductor presented in Figure 2c was established to study the electrical properties of β-Ga_2_O_3_ nanowires obtained by thermal oxidation. The current–voltage (I–V) characteristics were evaluated in dark and under UV light conditions for an MSM structure with and without an Ag catalyst at different voltages (Figure 4). The measurements performed at different voltages were found to be strongly influenced when different patterns of metal contacts were examined, which has a major impact on current control, as shown in Figure 4. The ratio of the photocurrent to dark current measured at 50 V for the Ga_2_O_3_ nanowire film on quartz (Figure 4) was almost 31.2 for the Ga_2_O_3_ film obtained with the Ag catalyst compared to 9.5 for Ag-free Ga_2_O_3_. 

Under a UV light intensity of 15 W/cm^2^, the MSM diode produced results comparable to the dark current. Furthermore, the results demonstrate that Ga_2_O_3_ on quartz obtained in the presence of 5 nm Ag as a catalyst enhanced the response of the UV photocurrent when the applied voltage increased (Table 2 and Figure 4). The results demonstrate that the photocurrent measured on the Ag/β-Ga_2_O_3_ nanowires was steady and about two–three orders of magnitude higher than the photocurrent measured on Ag-free nanowires (Table 2), which demonstrates the role played by an Ag catalyst in increasing the photocurrent response of β-Ga_2_O_3_ nanowires. The mechanism of β-Ga_2_O_3_ nanowire photodetection is attributed to several factors that mainly involve the absorption coefficient, photogenerated carriers, electrical transport properties, and the adsorption–desorption process of oxygen [12,15].

#### 3.2.2. Distance between Probes 

To compare the effects of the distance between two electrical probes, we used two different distances between probes to evaluate their effects. Decreasing the distance between the metal probes results in a decrease in resistivity, which is the reciprocal of conductivity ($\sigma =\frac{1}{\rho }$) [25]. Hence, decreasing the resistivity will increase conductance as the distance between the probes decreases. Consequently, as shown in Figure 4, the current improved as the distance became closer. 

Based on the electromagnetic theory (V = E. *l*), as the length increases, the electric field decreases and resistance increases [26]. As the electric field decreases, the movement of electrons begins to randomize because a weak electric field is not able to direct electrons in certain directions. In a weak electric field, the random movement of electrons results in an increase in the number of collisions between them; thus, it leads to an increase in the electrical resistance of the material. As the electric field increases, the flow of electrons improves; thus, decreasing the distance between the electrodes results in a decrease in the electrical resistance of the material. However, the leakage current of probes that were 1 mm apart was higher than the one where the probes were 3 mm apart. 

#### 3.2.3. Film Thickness

To measure the thickness of the film, the average height of nanowires was measured to estimate the average thickness. When the cross-section area is small, the space available for electrons to flow is reduced, and the movement of charges increases. As a result, electrical resistance increases. Because resistance is inversely proportional to the cross-sectional area (R α 1/A) [25], the electrical resistance of the material increases as the cross-sectional area decreases. Conversely, increasing the cross-sectional area results in a decrease in the number of collisions as the space between particles increases, and this leads to a decrease in the resistance of the material.

#### 3.2.4. Transient Photocurrent

**Figure 5:**
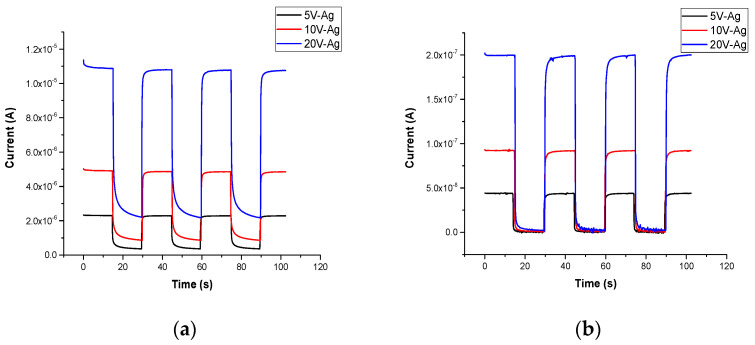
Transient response of the UV photodetector fabricated based on Au/β–Ga_2_O_3_/Au MSM at 5 V, 10 V, and 20 V. (**a**) The distance between the lines is <1 mm. (**b**) The distance between the circle probes is <3 mm.

The transient response of the photodetector is presented in Figure 5. By switching a UV light source on and off, a fast transient response was attained by Ga_2_O_3_ nanowires on quartz obtained in the presence of Ag catalyst because of higher carrier transport. Under UV illumination, a large increase in the photocurrent was obtained due to the increase in the photoconduction process by increasing carrier mobility by the adsorption and desorption of oxygen. However, when the UV light was switched off, the free electrons recombined with holes very quickly.

In Ga_2_O_3_ nanowires on bare quartz, the response of photodetector as the light being switched on or off was relatively weak because of the trapped surface state of the oxygen produced at the surface of Ga_2_O_3_ [9].

The average rise and fall times [12] of different applied voltages for Ag-free Ga_2_O_3_ and Ga_2_O_3_ obtained in the presence of Ag are summarized in Table 3. The average transient time was measured from 10% to 90% relative to the maximum photocurrent and vice versa for the rise and fall time, respectively.

Hence, Ag nanoparticles’ (NPs’) presence encourages faster detection. In Ga_2_O_3_ with an Ag catalyst, silver nanoparticles enhanced the adsorption and desorption of oxygen molecules that remained near the surface and contributed to the quick decrease in photocurrent upon UV illumination and fast photoresponse. The fall time of Ga_2_O_3_ in the presence of Ag is higher than that of the Ag-free Ga_2_O_3_. 

When UV light excites Ag nanoparticles, a dipole is induced in each nanoparticle [12]. The UV-induced dipole–dipole interaction in Ag nanoparticles directly influences each nanoparticle and its surroundings.

Table 4 shows the performance of the β-Ga_2_O_3_ device developed in our group compared to others. In this work, the ratio of photocurrent to dark current was 31.2 and was measured for the MSM Ga_2_O_3_ nanowires on quartz with 5 nm Ag catalyst at 5 V. A direct comparison to other applications is difficult due to the use of a single- or multi UV wavelength.

## 4. Conclusions

The β-Ga_2_O_3_ nanowire film obtained with and without a Ag catalyst for the UV photodetector has been demonstrated. Low-dimensional β-Ga_2_O_3_ nanowires have shown a high-performance compared to the UV photodetector, due to their quantum effects compared to the bulk materials. 

At different voltages, the patterns of the metal contacts had a significant impact on current control. The ratio of the photocurrent to dark current measured at 50 V was almost 31.2 and 9.5 for the Ga_2_O_3_ nanowire film on Ag-coated quartz and the one on bare quartz, respectively. For Ga_2_O_3_ NWs on Ag-coated quartz, the response of the UV photocurrent improved when the applied voltage increased. When the distance between the metal probes decreased, the current improved as the resistance of the material decreased, but the leakage current increased. A fast transient response was attained by Ga_2_O_3_ nanowires on Ag-coated quartz, while the photodetector response to light being on or off was relatively weak on Ga_2_O_3_ on bare quartz. 

The presence of a silver catalyst improved the growth mechanism and the electrical properties of Ga_2_O_3_ nanowires. Hence, this work recommends a UV photodetector that would offer a promising technique for mass production and to grow nanowires with high sensitivity and selectivity in harsh environments.

## Figures and Tables

**Table 1:** Summary of data that highlight the main differences between Ga_2_O_3_ nanowires on quartz without and with 5 nm Ag catalyst.

Material	Ga Only	5 nm Ag-Ga
NWs Avg. Length	5–60 µm	30–100 µm
NWs Avg. Diameter	300–868 nm	200 nm–1.00 µm
Density of NWs *	Less	High
Surface Morphology	Less uniform	Highly uniform

* The density was estimated visually based on SEM images.

**Table 2:** Current comparison of different applied voltages compared to the photocurrent and dark current of Ga_2_O_3_ on quartz with and without the presence of the silver catalyst.

Voltage (V)	Current, A			
	NoAg-Dark	NoAg-UV	Ag-Dark	Ag-UV
5	8.07 × 10^−11^	1.52 × 10^−8^	−1.23 × 10^−10^	1.27 × 10^−7^
	−4.64 × 10^−10^	2.20 × 10^−8^	2.39 × 10^−7^	3.15 × 10^−6^
10	1.23 × 10^−10^	3.36 × 10^−8^	5.15 × 10^−11^	2.76 × 10^−7^
	1.19 × 10^−9^	7.26 × 10^−8^	7.16 × 10^−7^	6.57 × 10^−6^
20	3.2 × 10^−10^	8.34 × 10^−8^	4.49 × 10^−10^	6.19 × 10^−7^
	2.75 × 10^−8^	2.69 × 10^−7^	2.15 × 10^−6^	1.44 × 10^−5^
50	1.4 × 10^−9^	2.23 × 10^−8^	5.65 × 10^−7^	4.63 × 10^−6^
	1.25 × 10^−9^	2.49 × 10^−7^	2.4 × 10^−9^	2.05 × 10^−6^

**Table 3:** Transient time of the photocurrent at different voltages of Ga_2_O_3_ on quartz under the presence of Ag catalyst with different distances of the metal contacts: 1 mm and 3 mm.

Transient Time	Distance (1 mm)			Distance (3 mm)		
	5 V	10 V	20 V	5 V	10 V	20 V
Rise Time	0.3	0.3	0.34	0.37	0.47	1.6
Fall Time	1.2	1.2	1.2	0.22	0.19	0.8

**Table 4:** Summary of β-Ga_2_O_3_ device performance of the present device and other previously reported UV PDs.

Device Structure	MSM	MSM	GR/oxide/GR	NW Network	MSM	MOS
**Fabrication Method**	Oxidation	MBE	LMBE	CVD	MOCVD	PECVD
**Electrode**	Cr/Au	Ti/Al		Au	Au	Au/Cr
**Light of Detection (nm)**	280–450	255	254	290–340	255–260	255
**I_Photo_/I_Dark_**	31.2	5.58 × 10^4^	82.88	50 @ 10V	-	<10^3^
**Year**	2022	2017	2017	2016	2015	2013
**Reference**	This work	[2]	[3]	[4]	[5]	[6]

## Data Availability

Not applicable.
